# miR-17-5p Downregulation Contributes to Paclitaxel Resistance of Lung Cancer Cells through Altering Beclin1 Expression

**DOI:** 10.1371/journal.pone.0095716

**Published:** 2014-04-22

**Authors:** Abhisek Chatterjee, Dhrubajyoti Chattopadhyay, Gopal Chakrabarti

**Affiliations:** Department of Biotechnology and Dr. B.C. Guha Centre for Genetic Engineering and Biotechnology, University of Calcutta, Kolkata, WB, India; IPMC, CNRS UMR 7275 UNS, France

## Abstract

Non- small- cell lung cancer (NSCLC) is one of the most leading causes of cancer-related deaths worldwide. Paclitaxel based combination therapies have long been used as a standard treatment in aggressive NSCLCs. But paclitaxel resistance has emerged as a major clinical problem in combating non-small-cell lung cancer and autophagy is one of the important mechanisms involved in this phenomenon. In this study, we used microRNA (miRNA) arrays to screen differentially expressed miRNAs between paclitaxel sensitive lung cancer cells A549 and its paclitaxel-resistant cell variant (A549-T24). We identified miR-17-5p was one of most significantly downregulated miRNAs in paclitaxel-resistant lung cancer cells compared to paclitaxel sensitive parental cells. We found that overexpression of miR-17-5p sensitized paclitaxel resistant lung cancer cells to paclitaxel induced apoptotic cell death. Moreover, in this report we demonstrated that miR-17-5p directly binds to the 3′-UTR of beclin 1 gene, one of the most important autophagy modulator. Overexpression of miR-17-5p into paclitaxel resistant lung cancer cells reduced beclin1 expression and a concordant decease in cellular autophagy. We also observed similar results in another paclitaxel resistant lung adenosquamous carcinoma cells (H596-TxR). Our results indicated that paclitaxel resistance of lung cancer is associated with downregulation of miR-17-5p expression which might cause upregulation of BECN1 expression.

## Introduction

Lung cancer is one of the most common malignancies and one of the leading causes of cancer related deaths in this world. Almost 85% of lung cancer cases belong to non- small- cell lung cancer (NSCLC) [1]. Paclitaxel based combination chemotherapies are now been considered as standard therapies for nearly all patients diagnosed with NSCLC [2]. Paclitaxel binds to the β- subunit of α- β tubulin heterodimer, stabilizes microtubule, reduces its dynamicity in the mitotic spindle, causes G_2_/M cell cycle arrest and drives the cancer cells to apoptotic death, activating spindle- mitotic check point [3]. Unfortunately, the clinical affectivity of paclitaxel is limited because some tumours show resistance or become resistant to it after repeated cycles of paclitaxel based chemotherapy which ultimately leads to relapse and poor prognosis. The most reported mechanisms of paclitaxel resistance involves upregulation of P-glycoprotein and related drug efflux pumps [4], [5], inadequate interaction with spindle microtubules due to posttranslational modification or altered expression of tubulin isotypes and microtubule-associated proteins [6]–[8] or functional change in cell signalling and cell survival pathways [9]–[12]. Recent studies show that autophagic induction by paclitaxel plays a major role in the development of paclitaxel resistance in tumor cells [13]–[15].

MicroRNAs, a highly conserved family of small, non- coding RNAs which recently emerged as novel class of gene expression modulators at posttranscriptional level [16]–[18]. This occurs through perfect or imperfect base pairing at the miRNA recognition elements (MREs) within the 3′ untranslated region (UTR) of target mRNAs, resulting in mRNA destabilization and translational repression [16], [19], [20]. Aberrant miRNA expression has been frequently observed in various human cancers including NSCLC [21], [22]. In recent years, attempts have been made to correlate dysregulation of particular miRNA expression with tumor responsiveness to chemotherapies, including paclitaxel [13], [23]–[26].

In this study, we were interested to examine the role of miRNAs in the development of paclitaxel resistance in lung cancer cells related to autophagy. We performed miRNA arrays to screen differentially expressed miRNAs between paclitaxel- sensitive (A549) and paclitaxel- resistant lung cancer cells (A549-T24). We identified that miR-17-5p was downregulated in paclitaxel resistant lung cancer cells (A549-T24 and H596-TxR) and its overexpression promoting paclitaxel induced cytotoxicity and apoptosis. Moreover, our data demonstrated that beclin1, one of the most important regulators of cellular autophagy, was a direct target of miR-17-5p in lung cancer cells.

Taken together all the findings we concluded that miR-17-5p played a critical role in the development of paclitaxel resistance by regulating cellular autophagy. Suppression of expression of miR-17-5p was associated with the upregulation of beclin1 expression and concordant autophagy which played a cyto-protective role and protected the cells from paclitaxel induced apoptosis and cell death.

## Materials and Methods

### Materials

Nutrient mixture Dulbecco’s modified eagle’s medium (supplemented with 1 mM L-glutamine), fetal bovine serum, penicillin-streptomycin, amphotericin B and 0.25% Trypsin-EDTA were purchased from GIBCO (Invitrogen). Paclitaxel was purchased from Sigma, USA. AnnexinV-FITC apoptosis kit was from Santa Cruz Biotechnology (Santa Cruz, CA, USA). JC-1and H2-DCFDA were purchased from Sigma, USA. Bradford protein estimation kit was purchased from Genei, India. All other chemicals and reagents were of analytical grade and were purchased from Sisco Research Laboratories, India.

### Cell Line and Cell Culture

Human non-small lung epithelial adenocarcinoma cell line Type II, A549, was obtained from the cell repository of National Centre for Cell Science (NCCS), Pune, India. Human lung adenosquamous carcinoma cell line NCI-H596 was obtained from American Type Culture Collection (ATCC). Both the cells were characterized by mt-rDNA sequencing for species identification, short tandem repeat profiling and isoenzyme analysis for cell line authentication and were confirmed to be negative for mycoplasma contamination by the repository. Both A549 and H596 cells were selected for resistance to paclitaxel (Sigma, USA) in a stepwise manner essentially as described [27], [28]. Briefly, A549 cells were initially exposed to 2 nM of paclitaxel and once normal growth was achieved the drug dose was increased in the multiples of two until a final concentration of 24 nM paclitaxel (A549-T24) was reached. For NCI-H596 cells which were a little tolerant to paclitaxel, were first exposed 5 nM of paclitaxel and maintained at this concentration until normal growth was reached. Thereafter the drug dose was enhanced in the multiples of three until a final dose of 15 nM of paclitaxel (H596-TxR) was achieved. Both the cells were maintained in Dulbecco’s Modified Eagle Medium (DMEM) supplemented with 1 mM L- glutamine, 10% fatal bovine serum, 3.7 gm/L NaHCO_3_, 100 µg/mL each of penicillin and streptomycin and 2.5 µg/mL amphotericin B. Cells were maintained at 37°C in a humidified atmosphere containing 5% CO_2._ Cells were grown upto 70–80% confluency in tissue culture flasks, then trypsinized with 0.25% trypsin- EDTA and divided into subsequent culture plates as required.

### miRNA Micro Array Expression Analysis

miRNA profiling of A549 and A549-T24 cells were done from Exiqon (Vedbaek, Denmark). Total RNA including miRNA was extracted from A549 and A549-T24 cells using Qiagen miRNeasy mini kit following the manufacturer protocol. The total RNA samples having adequate quality for analysis by miRCURY LNA miRNA microarray platform were labeled using the miRCURY LNA microRNA Hi-Power Labelling Kit, Hy3/Hy5 and pairs of sample were hybridized on the miRCURY LNA microRNA Array (6th gen - hsa, mmu & rno) and the array was read in Agilent G2505B Micro array scanner system in an ozone free environment. Only those miRNAs which were dysregulated by at least two fold (ΔLMR ≥2) were taken into consideration.

### Pre- miRNA Transfection

mirVana miRNA 17 mimic precursors (pre-miR-17-5p) and mirVana miRNA mimic negative control #1 (pre-miR-negative control) were purchased from Ambion. Pre-miRNAs were transfected into cell lines at ∼50% confluency at 100 nM concentration with Lipofectamine RNAiMAX (Invitrogen) transfection reagent. Forty-eight hours after transfection, the expression of miR-17-5p was detected by real-time PCR and the expression of BECN1 was tested by qRT-PCR and/or Western blotting.

### Quantitative Real-time PCR (qRT–PCR)

For miRNA expression analysis, qRT-PCR was done by using the TaqMan microRNA reverse transcription kit (Applied Biosystems) and TaqMan microRNA assays kit (Applied Biosystems) following the manufacturer’s protocols. U6 SnRNA served as the internal control.

To analyse the expression of BECN1, Bax, Bcl-2, LC3-II and glyceraldehyde-3-phosphate dehydrogenase (GAPDH) and caspase-3 (Table 1), cDNAs were synthesized from 1 µg of total RNA using SuperScript VILO cDNA Synthesis kit (Invitrogen). The cDNA was mixed with 2× DyNAmo ColorFlash SYBR Green qPCR Master Mix (Thermo Scientific) and various sets of gene-specific primers and then subjected to qRT-PCR quantification using the StepOne- Plus real time PCR system (Applied Biosystems). Gene expression was calculated relative to GAPDH (for BECN1, Bcl-2, Bax, LC3-II, caspase-3 etc) or U6 SnRNA (for miR-17-5p) using the comparative cycle time (Ct) method (2^−ΔΔCt^ method) [29], [30].

**Table 1:** Primer sequences used for Real- time PCR.

*Gene name*	*Forward Primer sequence (5′-3′)*	*Reverse Primer sequence (5′-3′)*
*Beclin1*	CAAGATCCTGGACCGTGTCA	TGGCACTTTCTGTGGACATCA
*LC3-II*	GAGAAGCAGCTTCCTGTTCTGG	GTGTCCGTTCACCAACAGGAAG
*Caspase-3*	GAGCTGCCTGTAACTTG′	ACCTTTAGAACATTTCCACT
*Bcl-2*	TTGGATCAGGGAGTTGGAAG	TGTCCCTACCAACCAGAAGG
*Bax*	GGACGAACTGGACAGTAACATGG	GCAAAGTAGAAAAGGGCGACAAC
*p53*	GTTCCGAGAGCTGAATGAGG	TTATGGCGGGAGGTAGACTG
*GAPDH*	CACCATGGAGAAGGCTGGGGCTC	GCCCAGGATGCCCTTGAGGG

### Cell Proliferation Inhibition Assay (MTT Assay)

A549-T24 and H596-TxR cells, either transfected with 100 nM pre-miR-17-5p (T24-miR-17-5p and TxR-miR-17-5p respectively) or with 100 nM pre-miR-negative control RNA (T24-miR-NC and TxR-miR-NC respectively) were plated in 96-well culture plates (1×10^4^ cells per well). After 24 h incubation, the media was replaced with fresh media and the cells were treated with different concentrations of paclitaxel for another 24 h. MTT (5 mg/mL) dissolved in PBS and filter sterilized, then 20 µL of the prepared solution was added to each well. This was incubated until purple precipitate was visible. Subsequently 100 µL of Triton-X100 was added to each well and incubated in darkness for 2 h at room temperature. The absorbance was measured on an ELISA reader (MultiskanEX, Lab systems, Helsinki, Finland) at a test wavelength of 570 nm and a reference wavelength of 650 nm. Data were calculated as the percentage of inhibition by the following formula:
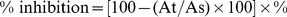
(1)


A_t_ and A_s_ indicated the absorbance of the test substances and solvent control, respectively [31].

### Trypan Blue Exclusion Assay for Cell Viability

Trypan blue exclusion assay [32] was used to determine the number of viable and dead cells following pre-miR-17-5p transfection and subsequent paclitaxel treatment. Briefly, T24-miR-17-5p or T24-miR-NC cells were plated in 6-well culture plates (5×10^4^ cells per well). Then cells were treated with 24 nM and 50 nM paclitaxel for another 24 h. After 24 h cells were harvested through trypsinization, washed twice with 1X PBS and then stained with 0.4% trypan blue in PBS. Cells were then prepared for analysis by flow cytometry.

### Construction of Luciferase Reporter Constructs and Luciferase Activity Assay

The 3′UTR-luciferase reporter constructs containing the 3′UTR of BECN1 with or without miR-17-5p binding site were PCR amplified from total cDNAs prepared from total RNA obtained from A549-T24 cells. The PCR products were cloned into *pCI-neo-RL-luc* reporter vector (A generous gift from Dr. SN Bhattacharyya, IICB, Kolkata, India.) between *Xba*I and *Not*I restriction sites, immediately downstream of the renilla luciferase gene. All the luciferase constructs were sequence verified. Cells were transiently co- transfected with renilla luciferase reporter plasmids (*pCI-neo-RL-Bec-3*′*UTR-wt* or *pCI-neo-RL-Bec-3*′*UTR-mut*), firefly luciferase plasmid (*pGL3-FF*) and pre-miR-17-5p precursor and/or anti-miR-17-5p or pre-miR-negative control precursor RNA using lipofectamine2000 transfection reagent following manufacturer’s protocol. After 48 h of transfection, cells were harvested and lysed with passive lysis buffer (Promega). The luciferase activities in the cellular extracts were determined by using Promega dual luciferase reporter assay kit on a VICTOR X3 Plate Reader system (PerkinElmer). The relative luciferase activities were calculated by the ratio of Renilla luc/Firefly luc activity and normalized to that of the control cells and fold repression was calculated. pGL3-FF vector was used as the internal control.

### Detection of Acidic Vesicular Organelles (AVOs)

To observe the reduction in AVOs formation, T24-miR-17-5p and T24-miR-NC cells were seeded on cover slips. After 48 h of transfection, cells were stained with acridine orange (AO) (1 µg/ml) at 37°C in the dark for 15 min, then washed twice with PBS. Images of AO staining were visualized immediately using fluorescence microscope (OLYMPUS IX70, Japan). The cytoplasm and nucleus of stained cells fluoresced bright green, whereas the acidic autophagic vacuoles fluoresced bright red. Similar experiments were also performed with A549 cells.

To quantify the change in number of acidic vesicles (AVOs) in A549, T24-miR-NC and T24-miR-17-5p cells, they were stained with AO (1 µg/ml) in PBS at 37°C for 15 min, the cells were harvested, washed twice in PBS and resuspended in 500 µl PBS and then analysed immediately by flow cytometry assay. The flow cytometric data was analysed with CellQuest analysis software (Becton Dickinson) [33].

### Labelling of Autophagic Vacuoles with Monodansylcadaverine

A549-T24 cells were transfected either with pre-miR-17-5p (100 nM) or with pre-miR- negative control RNA (100 nM), seeded on coverslips and kept for another 48 h. Cells were incubated with 50 µM monodansylcadaverine for 10 min at 37°C in PBS and images were taken by fluorescence microscope (OLYMPUS IX70, Japan). Moreover, for quantitative analysis of autophagosome formation same samples were trypsinized and analysed by flow cytometry assay [34].

### Flow Cytometric Analysis for Apoptotic Cells

T24-miR-NC and T24-miR-17-5p cells were treated with 24 nM and 50 nM paclitaxel for 24 h. Approximately 1×10^5^ cells were then stained for 15 min at room temperature in the dark with FITC-conjugated annexinV (1 µg/ml) and propidium iodide (PI) (0.5 µg/ml) in a Ca^2+^-enriched binding buffer and analyzed by a two color flow cytometric assay. AnnexinV and PI emissions were detected in the FL1 and FL2 channels of a FACSCalibur flow cytometer (Becton-Dickinson, USA) respectively [31]. The data were analysed using CellQuest program from Becton-Dickinson.

### Determination of Mitochondrial Membrane Potential (ΔΨ)

To evaluate the mitochondrial membrane potential (ΔΨ), 5,5′,6,6′-tetrachloro-1,1′,3,3′-tetraethylbenzimidazolylcarbocyanine iodide (JC-1), a sensitive fluorescent probe for ΔΨ was used [35]. T24-miR-NC and T24-miR-17-5p cells were treated with 24 nM and 50 nM paclitaxel for 24 h. Cells were then harvested, washed twice with PBS, stained with 5 µM JC-1 for 30 min at 37°C in the dark. Cells were rinsed with PBS twice, resuspended in 500 µl PBS and instantly assessed for red fluorescence (JC-1) with FACSCalibur flow cytometer (Becton-Dickinson, USA).

### Measurement of Reactive Oxygen Species (ROS)

ROS levels were determined using the fluorescent marker 2′,7′- dichlorodihydrofluorescein diacetate (DCFH-DA) [36]. Briefly, T24-miR-NC and T24-miR-17-5p cells were treated with 24 nM and 50 nM paclitaxel for 24 h. Cells were trypsinized, washed with PBS and incubated with 10 mM DCFH-DA for 30 min in the dark at room temperature and the shift in the green fluorescence intensity, as detected in FL1 channel, was followed by FACSCalibur flow cytometer (Becton-Dickinson, USA) and the data was analysed with CellQuest analysis software (Becton-Dickinson, USA).

### Statistical Analysis

qRT-PCR reactions were run in triplicate for each sample and repeated at least 3 times and the data were statistically analyzed with Student’s ‘t-test’ or Wilcoxon rank sum test. IC_50_ data from the MTT assay were analyzed with Wilcoxon rank sum test. All data were shown as the means ± S.E. (Standard error). Two measurements were statistically significant if the corresponding p value was <0.05.

## Results

### Profiles of miRNAs in Paclitaxel- sensitive and Resistant Lung Cancer Cells

**Figure 1:**
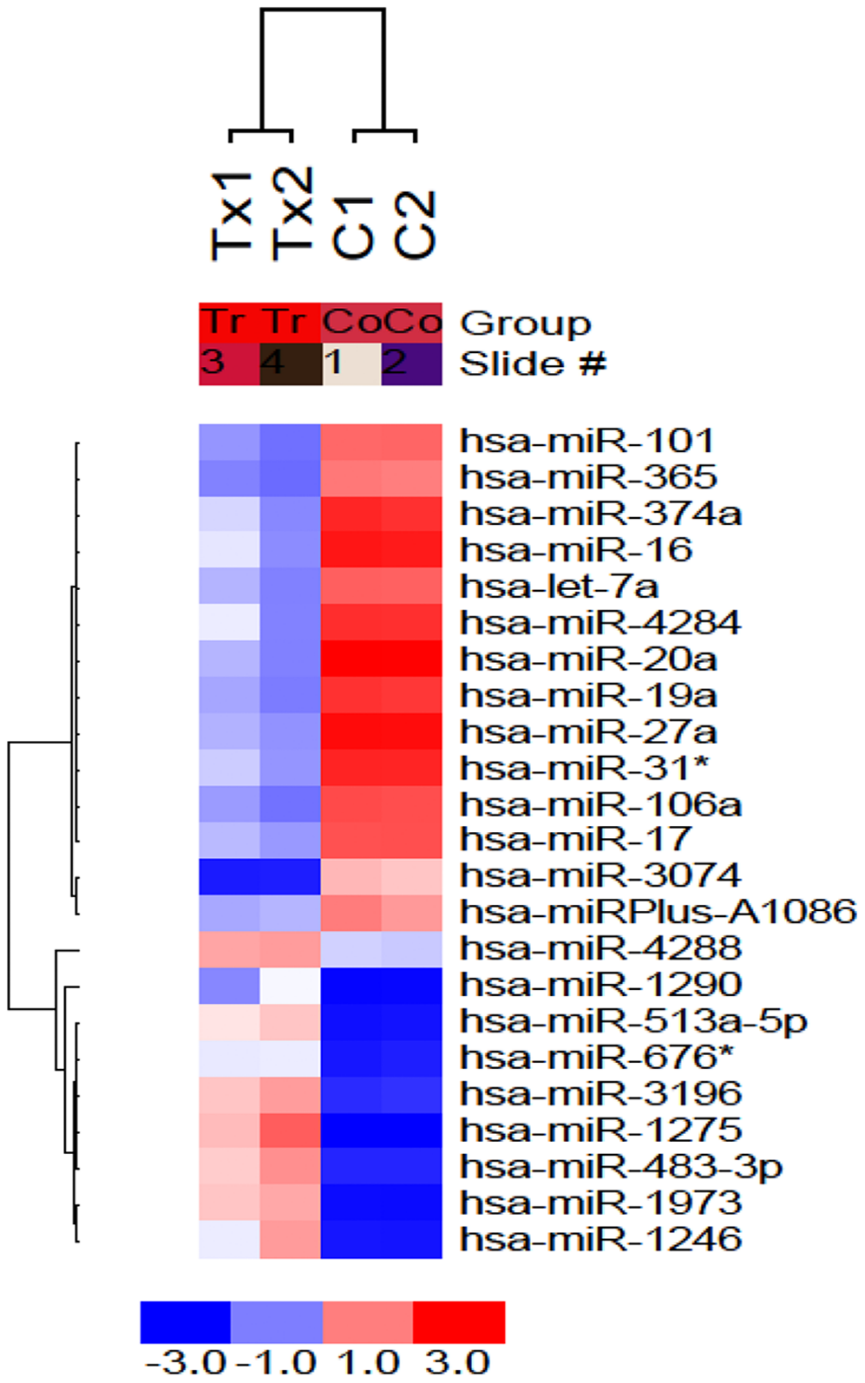
Micro-RNA profiles of paclitaxel resistant and paclitaxel sensitive A549 cells. miRNA array profiles of paclitaxel sensitive (C1 and C2) and resistant (Tx1 and Tx2) lung cancer cell lines was shown. Supervised hierarchical clustering of cell lines based on their differential miRNAs expression with ΔLMR≥2 between the two groups was exhibited. Each column represents a cell line and each row a probe set. The heat map indicates high (red) or low (blue) level of expression relative to the mean as per the scale shown in the figure.

We prepared paclitaxel-resistant lung non-small cancer cell (A549-T24) from paclitaxel sensitive cells by continuous exposure of A549 cells to paclitaxel (Fig. S1A). To search for the critical miRNAs involved in the development of paclitaxel resistance, total RNA, extracted from lung cancer cells (A549) and their paclitaxel resistant variant (A549-T24), were sent to Exiqon, Denmark for miRNA array profiling. From the array results it was identified that 23 miRNAs were differentially expressed in A549-T24 cells than A549 cells (Fig. 1).

**Figure 2:**
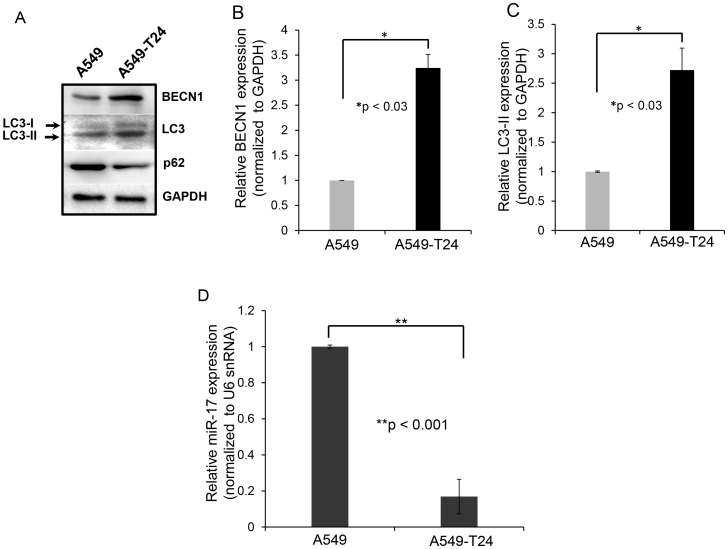
A549-T24 cells exhibit heightened level of autophagy with downregulated miR-17-5p expression compared to paclitaxel sensitive A549 cells. (A) Expression status of certain autophagic marker proteins BECN1, MAP-LC3, p62 and GAPDH (loading control) were measured by Western blotting. (B–C) Relative BECN1 and LC3-II mRNA expression levels were quantified by qRT-PCR analysis in A549 and A549-T24 cells, *bars* represent mean ± S.E. (*p<0.03 vs control, where n = 3) (D) Downregulation of miR-17-5p expression in paclitaxel resistant lung cancer cells (A549-T24) compared to A549 cells. Taqman qRT-PCR was performed to detect the relative levels of miR-17-5p in A549 and A549-T24 cells. Results were normalized to snU6 expression level and represented as mean ± S.E. from three independent replicates. (**p<0.001 vs control, n = 3).

To identify the target genes of these differentially expressed miRNAs, we searched miRNA target prediction databases miRBase, microRNA.org and TargetScanHuman 6.2 and we identified miR-17-5p could probably have a role in autophagy by targeting autophagy related protein beclin 1 (BECN1) by binding directly to its 3′ UTR region between position 135 to 141. It is already reported that induction of autophagy by paclitaxel treatment plays a major role in the development of paclitaxel resistance in tumor cells with upregulation of BECN1 [13]-[15]. We observed increased level of autophagy as indicated by upregulation of BECN1 and increased LC3-I to LC3-II conversion and downregulation of p62 expression in A549-T24 cells compared to A549 cells (Fig. 2A). We also measured relative mRNA levels of BECN1 and LC3-II (Fig. 2B and 2C) and found both these mRNAs were upregulated in A549-T24 cells compared to A549 cells. To extend our finding we prepared another paclitaxel resistant lung cancer cell line H596-TxR from paclitaxel sensitive NCI- H596 cells *in vitro* (Fig. S1B) and examined the status of BECN1 and LC3 in this resistant lung cancer cell line (H596-TxR). We found similar upregulation of BECN1 and increased LC3-I to LC3-II conversion in paclitaxel resistant H596 cells (H596-TxR) compared to parental H596 cells (Fig. S2A). Furthermore, analysis of relative mRNA levels of BECN1 and LC3-II (Fig. S2B and Fig. S2C) also confirmed significant upregulation of cellular autophagy in H596-TxR cells compared to H596 cells.

### miRNA-17-5p is Downregulated in Paclitaxel- Resistant Lung Cancer Cells

We further validated the array data for miR-17-5p by Taqman qRT-PCR. Using Taqman probe- based qRT- PCR assay, we compared the expression level of miR-17-5p in paclitaxel sensitive and resistant A549 cells. Fig. 2D shows a ∼7.2 fold downregulation in the relative miR-17-5p expression level in A549-T24 cells compared to A549 cells, validating the micro-array results. We also estimated the expression level of miR-17-5p in H596-TxR cells and compared it with that of H596 cells. We found that H596-TxR cells exhibited almost ∼2.62 fold downregulation of relative miR-17-5p expression compared to that of paclitaxel sensitive H596 cells (Fig. S2D) indicating association between miR-17-5p and paclitaxel resistance was not cell line specific.

### Sensitivity to Paclitaxel is Modulated by Overexpression of miR-17-5p *in vitro*

**Figure 3:**
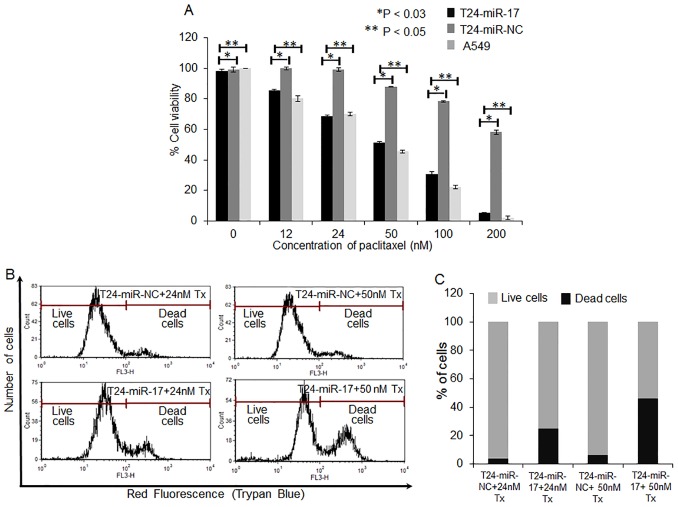
miR-17-5p is functionally involved in the paclitaxel response to A549 cells. (A) A549-T24 cells were either transfected with 100 nM pre-miR-negative control (T24-miR-NC) or pre-miR-17-5p (T24-miR-17-5p) precursor RNA and were seeded into 96 well plates at a density of 1×10^4^ cells per well. After 24 h, cells were treated with 0, 12, 24, 50, 100, 200 nM paclitaxel for another 24 h. The cell viability was assessed by MTT assay. Data are presented as % of cell viability measured in cells treated with paclitaxel. *Columns*, mean of three independent experiments; *bars*, mean ±S.E. (*p<0.05 vs negative control, p<0.03 vs A549 control, where n = 4). (B–C) Cells (T24-miR-NC and T24-miR-17-5p) were subsequently treated with 24 nM and 50 nM paclitaxel for 24 h and are subjected to FACS analysis after being stained by Trypan blue. Histogram represents the Red fluorescence intensity (FL3-H) vs. counts plot where dose dependent increase of cell death occurs. The results represent the best of data collected from three experiments with similar results.

To investigate whether miR-17-5p overexpression sensitized A549-T24 cells to paclitaxel, we transfected A549-T24 cells with 100 nM pre-miR-17-5p (T24-miR-17-5p) or 100 nM pre-miR- negative control RNA (T24-miR-NC) and 24 h following transfection cells were treated with different doses of paclitaxel (0–200 nM). It was observed that compared with the negative control (T24-miR-NC), overexpression of miR-17-5p significantly sensitized the A549- T24 cells to paclitaxel (Fig. 3A, *p<0.05* and *p<0.03*). For example, T24-miR-17-5p cells exhibited almost 86%, 51%, 31% and 6% cell viability when cells were treated with 12 nM, 50 nM, 100 nM and 200 nM paclitaxel for 24 h respectively. Under similar conditions T24-miR-NC cells showed almost 99%, 89%, 78% and 58% viability. Similar results were obtained when we overexpresed 100 nM pre-miR-17-5p into H596-TxR cells (TxR-miR-17-5p) and treated the cells with similar doses of paclitaxel (0–200 nM) for 24 h. It was observed that compared to the negative control (TxR-miR-NC), TxR-miR-17-5p cells exhibited much lower cell viability when treated with increasing concentrations of paclitaxel. Complete dose response curves are shown in Fig. S3A.

This loss of cell viability by miR-17-5p overexpression and subsequent paclitaxel treatment was further assessed by trypan blue dye exclusion assay using flow cytometer (Fig. 3B and 3C, *p<0.01*) with A549-T24 cells. Live cells possess intact cell membranes that exclude trypan blue, however, dead cells do not and eventually take up trypan blue. Fig. 3B and 3C shows that with miR-17-5p overexpression and subsequent paclitaxel treatment (24 and 50 nM) for 24 h, residual cell viability of T24-miR-17-5p cells was decreased compared to the respective negative controls. For example, while miR-17-5p overexpression and subsequent treatment with 24 nM and 50 nM paclitaxel for 24 h caused almost 25% and 46% cell death, corresponding negative controls exhibited only 4% and 6% cell death, respectively. These results indicated that miR-17-5p overexpression plays a key role in sensitizing paclitaxel resistant A549-T24 cells to paclitaxel.

### Beclin 1 is a Direct Target of miR-17-5p in Lung Cancer Cells

**Figure 4:**
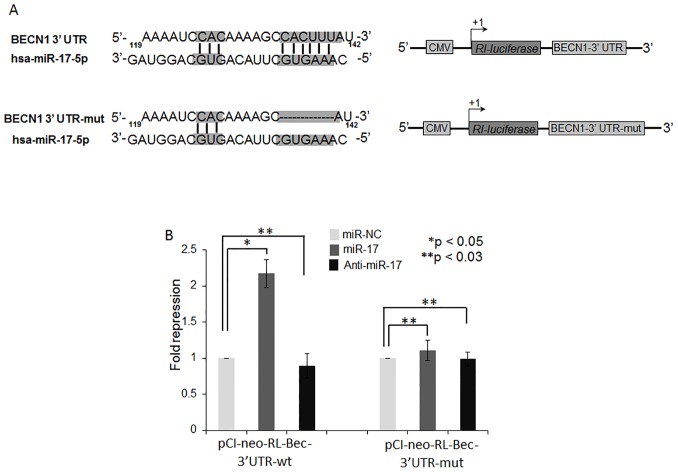
BECN1 is a direct target of miR-17-5p. (A) Schematic representation of the 3′- UTR of human BECN1 transcript. Predicted miR-17-5p binding site was depicted. The numbers (+135–141) represented the nucleotides that were predicted to base pair with the miR-17-5p seed sequence. (B) miR-17-5p directly binds to the 3′ UTR of BECN1 gene in A549-T24 cells. A549-T24 cells were co- transfected with renilla luciferase reporter plasmids (*pCI-neo-RL-Bec-3*′*UTR-wt* or *pCI-neo-RL-Bec-3*′*UTR-mut*), firefly luciferase plasmids (pGL3-FF) and pre-miR-17-5p precursor or anti-miR-17-5p or pre-miR-negative control precursor RNA using lipofectamine2000 transfection reagent. After 48 h, cells were harvested and lysed with passive lysis buffer. Luciferase activity was measured by using Promega dual luciferase reporter assay kit. The results were represented as relative fold repression (Renilla luc/Firefly luc activity) compared to control cells. (*p<0.05, **p<0.03 vs control, n = 4).

To determine whether miR-17-5p directly binds to the 3′- UTR region of BECN1 mRNA, we constructed 3′- UTR reporters of BECN1 containing putative miR-17-5p binding site and corresponding mutant construct, lacking miR-17-5p binding site, downstream of the luciferase reporter gene (Fig. 4A). Co-transfection of pre-miR-17-5p with BECN1 wild- type reporter construct in A549-T24 cells greatly repressed renilla luciferase activity (Fig. 4B), while co-transfection with mutant reporter construct showed no significant change in relative luciferase activity with that of control (Fig. 4B). Moreover, when A549-T24 cells were co transfected with anti-miR-17-5p (100 nM) and 3′- UTR reporters of BECN1, with or without putative miR-17-5p binding site, no significant decrease in relative luciferase activity was observed (Fig. 4B). These results collectively confirmed that BECN1 was a direct target of miR-17-5p in lung cancer cells.

### Overexpression of miR-17-5p in Paclitaxel Resistant Lung Cancer Cells Leads to Beclin 1 Downregulation

**Figure 5:**
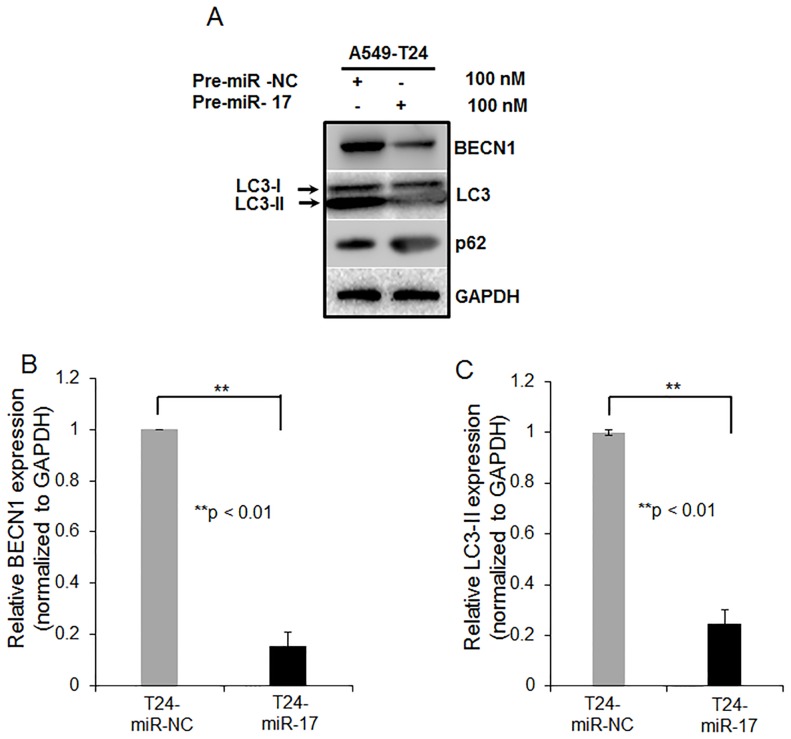
miR-17-5p modulates BECN1 expression in A549-T24 cells. (A) A549-T24 cells were transfected either with 100 nM pre-miR-negative control (T24-miR-NC) or pre-miR-17-5p (T24-miR-17-5p) precursor RNA. After 24 h, cell lysates were prepared for Western blotting with antibody against BECN1, MAP-LC3, p62 and GAPDH was used as loading control. (B–C) Relative BECN1 and LC3-II mRNA expression levels were quantified by qRT-PCR analysis in T24-miR-NC and T24-miR-17-5p cells, *bars* represent mean ± S.E. from three independent experiments (**p<0.01 vs control, where n = 3).

To experimentally validate target prediction, we assessed the protein levels of BECN1 following miR-17-5p overexpression into both A549-T24 and H596-TxR cells. Fig. 5A shows, overexpression of miR-17-5p significantly decreased BECN1 expression as compared to the negative control (T24-miR- NC). This was further confirmed by the assessment of mRNA levels of BECN1 by qRT-PCR. We found, miR-17-5p overexpression reduced BECN1 mRNA level by ∼5.6 fold (*p<0.01*) in A549-T24 cells as compared to the negative control. We tested the expression levels of two other autophagic markers, LC3-II and p62 which are downstream of BECN1 by Western blotting. Overexpression of miR-17-5p into A549-T24 cells reduced conversion of LC3-I to LC3-II (Fig. 5A, second blot and Fig. 5C) and increased level of p62 (Fig. 5A, third blot). In case of H596-TxR cells, we also found that overexpression of miR-17-5p resulted in concordant downregulation of BECN1 and LC3-II in H596-TxR cells (Fig. S3B–D). All these data collectively proved that overexpression of miR-17-5p into paclitaxel resistant lung cancer cells caused reduction in cellular autophagy by directly targeting autophagy related protein BECN1.

### Overexpression of miR-17-5p Inhibits Autophagy in Paclitaxel Resistant A549-T24 Cells

**Figure 6:**
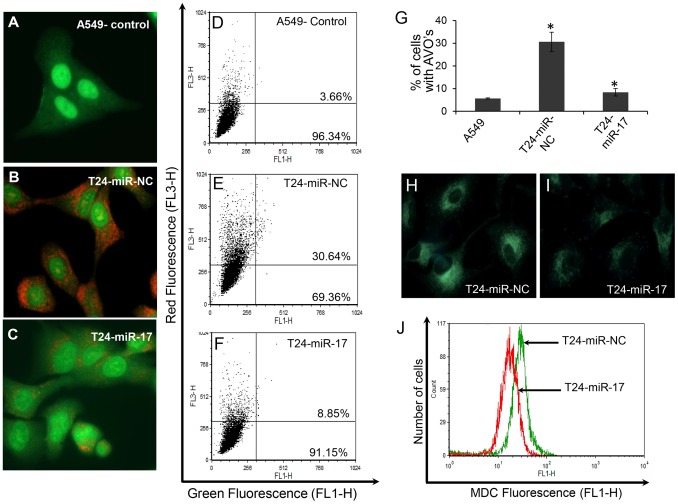
Detection of suppression of autophagy following miR-17-5p overexpression into paclitaxel resistant A549-T24 cells. T24-miR-NC and T24-miR-17-5p cells were stained with AO for AVO observation under fluorescence microscope (B and C). AO staining of A549 served as the control (A). (D–F) To quantitate change in % of cells developing AVO following miR-17-5p overexpression into A549-T24 cells, flow- cytometric estimation of AVOs were done in A549, T24-miR-NC and T24-miR-17-5p cells respectively. (G) Quantitation of mean AVO positive cells in all three cell lines. Data presented are the mean ± S.E. (*p<0.05 vs control, where n = 3). (H- I) MDC staining of T24-miR-NC and T24-miR-17-5p cells under fluorescence microscope. (J) Quantitation of change in autophagic vacuole formation in T24-miR-17-5p compared to T24-miR-NC cells by MDC staining and Flow- cytometry. Histogram represents relative MDC fluorescence intensity (FL1-H) vs cell counts plot.

Paclitaxel induces autophagy in cancer cells [13]–[15]. Tumour cells use this cytoprotective autophagy as a defence from apoptotic cell death which in turn contributes to development of paclitaxel resistance. During authophagy, autophagosomes fuse with lysosomes to form autophagolysosomes which are acidic vacuoles (AVO) that bind acridine-orange giving red fluorescence and can be easily assessed by fluorescence microscopy and flow cytometer. Fig. 6A–B showed while paclitaxel sensitive A549 cells exhibited almost no red vesicles (Fig. 6A), large number of red fluorescent vesicles were observed in the cytoplasm of T24-miR-NC cells (Fig. 6B). However, when A549-T24 cells were transfected with miR-17-5p (T24-miR-17-5p cells), appearance of red AVO’s reduced significantly (Fig. 6C). These results were reconfirmed by flow cytometric analysis (Fig. 6D–G). We observed that T24-miR-NC cells showed higher levels of autophagic flux compared to A549 cells (Fig. 6D compared to Fig. 6E). However, when A549-T24 cells were overexpressed with miR-17-5p, formation of AVO’s reduced significantly as compared to T24-miR-NC cells (Fig. 6F compared to Fig. 6E). Fig. 6G shows quantitative representation of AVOs in cells which were calculated from flow cytometer results.

To selectively stain autophagic vacuoles, we used monodansylcadaverine (MDC) staining. MDC accumulates in mature autophagic vacuoles, but not in early endosomal compartments, which are also acidic [13]. We found that overexpression of miR-17-5p in A549-T24 cells resulted in the decrease in MDC mean fluorescence as compared to negative control (T24-miR- NC) (Fig. 6H compared to Fig. 6I and Fig. 6J) indicating reduction in autophagosome count. All these data collectively demonstrated that overexpression of miR-17-5p resulted in inhibition of autophagy in paclitaxel-resistant lung cancer cells by targeting BECN1. We also observed reduction in autophagy following miR-17-5p overexpression into H596-TxR cells (data not shown).

### Overexpression of miR-17-5p Induced Apoptosis in Paclitaxel-resistant Lung Cancer Cells

**Figure 7:**
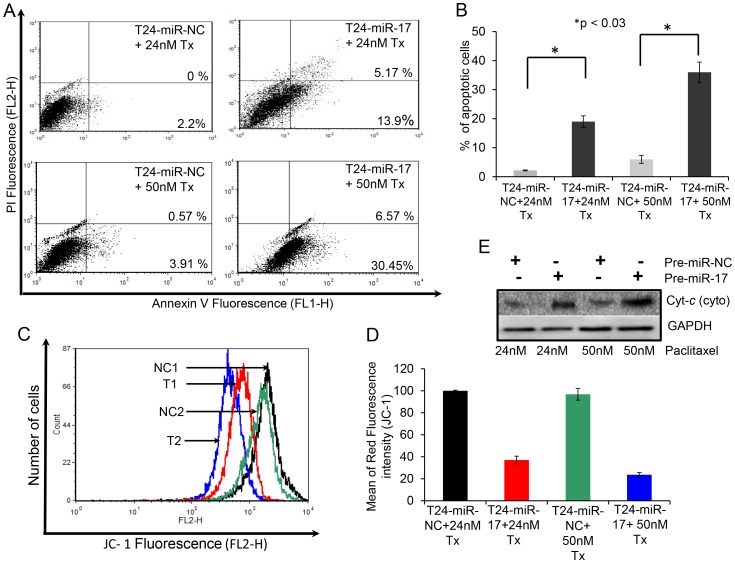
miR-17-5p overexpression and subsequent paclitaxel treatment induced mitochondrial pathway mediated apoptosis in A549-T24 cells. (A–B) Overexpression of miR-17-5p induced apoptosis in paclitaxel resistant lung cancer cells. (A) T24-miR-NC or T24-miR-17-5p cells were treated either with 24 nM or 50 nM paclitaxel for another 24 h. Cells were then harvested for apoptosis analysis by annexinV- FITC/PI staining and flowcytometry. The % of early apoptotic cells (annexinV-FITC positive/PI negative cells) and late apoptotic cells (annexinV-FITC positive/PI positive cells) were determined. The results represented are the best of data collected from three independent experiments with similar results. (C–D) miR-17-5p overexpression and subsequent treatment with paclitaxel induced collapse of mitochondrial membrane potential in A549-T24. T24-miR-NC and T24-miR-17-5p cells were treated with 24 nM and 50 nM paclitaxel for 24 h. Cells were harvested and stained with JC-1 fluorescent dye. Histogram represents drop in red fluorescence (FL2-H) intensity vs. cell counts plot where mitochondrial membrane potential decreases following miR-17-5p overexpression and subsequent paclitaxel treatment. NC1, T1, NC2 and T2 represents T24-miR-NC cells treated with 24 nM paclitaxel, T24-miR-17-5p cells treated with 24 nM paclitaxel, T24-miR-NC cells treated with 50 nM paclitaxel and T24-miR-17-5p cells treated with 50 nM paclitaxel respectively. The results represent the best of data collected from three independent experiments with similar results. (E) Western blot analysis to detect the release of the cytochrome-*c* in the cytosol from mitochondria in A549 T24 cells following miR-17-5p overexpression and subsequent paclitaxel treatment.

Paclitaxel exerts its cytotoxic effect by inducing apoptosis [15]. However, in drug resistant cancer, tumor cells overcome this cytotoxic effect of paclitaxel and become resistant to apoptosis. We already examined whether the inhibition of autophagy by miR-17-5p over expression sensitizes lung cancer cells to paclitaxel-induced cell death by MTT assay (Fig. 3A and Fig. S3A). Next, we examined whether overexpression of miR-17-5p and subsequent treatment with paclitaxel was capable of inducing apoptotic death in drug resistant lung cancer cells. A549-T24 cells, either transfected with 100 nM pre-miR-17-5p (T24-miR-17-5p) or with 100 nM pre-miR-negative control RNA (T24-miR-NC) were treated either with 24 nM or with 50 nM paclitaxel for 24 h. Then cells were doubly labelled with FITC-AnnexinV and PI and then analyzed by flow cytometer. Fig. 7A revealed that significantly more apoptotic cells were detected upon paclitaxel treatment in T24-miR-17-5p cells compared to T24- miR-NC cells. The number of apoptotic cells were 2.2% and 4.48% for T24-miR-NC, treated with 24 nM or 50 nM paclitaxel for 24 h and 19.07% and 37.02% in case of T24-miR-17-5p cells treated with 24 nM and 50 nM paclitaxel for 24 h respectively (Fig. 7A and 7B). Moreover, consistent with the above results, when paclitaxel induced apoptosis following miR-17-5p overexpression in H596-TxR cells was determined, it was observed that miR-17-5p overexpression increased apoptosis in H596-TxR cells (Fig. S4A and S4B, *p<0.05*). For example, while TxR-miR-NC cells showed only 0.79% and 3.64% annexin positive cells when treated with 24 nM and 50 nM paclitaxel for 24 h respectively, transfection with pre-miR-17-5p and subsequent paclitaxel treatment resulted in significant increase in the number of apoptotic cells (11.38% and 30.6% respectively) under similar condition. Taken together it appears that overexpression of miR-17-5p and subsequent paclitaxel treatment induces apoptotic cell death in lung cancer cells.

### Overexpression of miR-17-5p Induced Disruption of Mitochondrial Membrane Potential and Release of Cytochrome-*c* from Mitochondria in A549-T24 Cells

Mitochondrial pathway mediated apoptosis is often associated with the collapse of membrane potential ΔΨ as a result of depolarization and leakiness of the inner mitochondrial membrane. Therefore, to examine whether mitochondrial membrane integrity is damaged by miR-17-5p overexpression and subsequent paclitaxel treatment in A549-T24 cells, change in ΔΨ was measured using JC-1 dye. At low ΔΨ, JC-1 exists mainly in a monomeric form, which emits green fluorescence whereas it form aggregates and emits red fluorescence at high ΔΨ which easily could be followed by flow cytometer. As shown in Fig. 7C and 7D, inhibition of autophagy by miR-17-5p overexpression and subsequent paclitaxel treatment of A549-T24 cells induced collapse of ΔΨ as observed by drop in red fluorescence intensity (JC-1). These data suggested that loss of mitochondrial membrane potential might be an early event of paclitaxel induced cell death. Thereafter, we checked whether drop in mitochondrial membrane potential had any effect on the release of cytochrome-*c* in the cytosol from mitochondria. The amount of cytochrome-*c* in the cytosol of T24-miR-NC and T24-miR-17-5p cells were analysed by Western blotting using anti-cytochrome-*c* antibody. Fig. 7E shows, compared to the respective negative controls, with miR-17-5p overexpression and subsequent paclitaxel treatment amount of cytochrome-*c* increased in the cytosol of T24-miR-NC cells. Moreover, we also checked the status of cytosolic cytochrome-*c* in TxR-miR-17-5p cells following treatment with 50 nM of paclitaxel for 24 h (Fig. S6A). We found that miR-17-5p overexpression and subsequent treatment with paclitaxel resulted in release of cytochrome-*c* into the cytosol from mitochondria in H596-TxR cells (Fig. S6A).

### Overexpression of miR-17-5p Caused the Change in Expression of Pro-apoptotic and Anti- apoptotic Proteins in Paclitaxel Resistant Lung Cancer Cells

**Figure 8:**
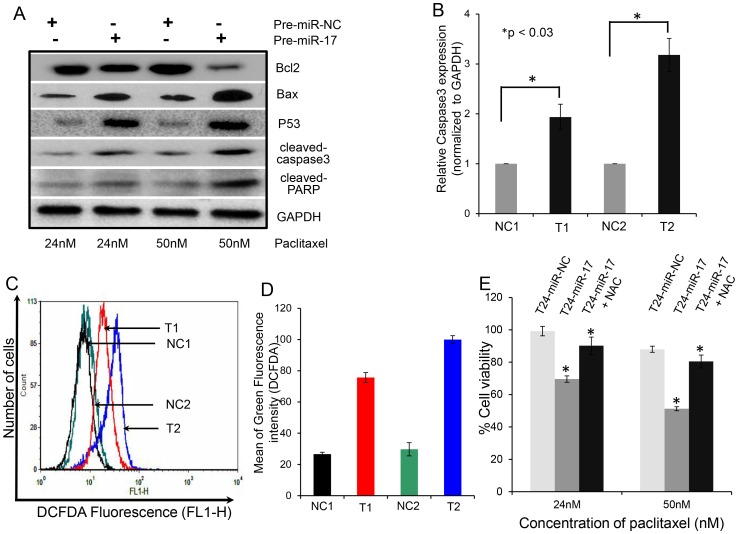
miR-17-5p overexpression and subsequent paclitaxel treatment caused change in the expression of pro-apoptotic and anti- apoptotic proteins and also stimulated ROS generation in A549-T24 cells. (A) Western Blot analysis of apoptotic marker proteins. Western Blot analysis of change in expression of pro- and anti-apoptotic proteins (Bax, Bcl-2, P53, cleaved caspase3, cleaved PARP) in A549-T24 cells following miR-17-5p overexpression and paclitaxel treatment. Briefly, T24-miR-NC or T24-miR-17-5p cells were treated with 24 nM or 50 nM paclitaxel for 24 h and cell lysates were prepared for Western blotting using antibodies against Bax, Bcl-2, P53, cleaved caspase3, cleaved PARP. GAPDH served as the loading control. (B) Measurement of relative caspase3 mRNA level by qRT- PCR. Relative caspase3 mRNA was estimated by qRT-PCR in T24-miR-17-5p cells compared to T24-miR-NC cells following 24 nM or 50 nM paclitaxel treatment for 24 h. (C–D) miR-17-5p overexpression and subsequent paclitaxel treatment stimulated ROS generation in A549-T24 cells. T24-miR-NC or T24-miR-17-5p cells were treated with 24 nM or 50 nM paclitaxel for 24 h. ROS generation were estimated by staining the H2-DCFDA staining and flow cytometry. NC1, T1, NC2 and T2 represent the same as mentioned earlier. (E) Amelioration of paclitaxel induced cytotoxicity following miR-17-5p overexpression in A549-T24 cells by NAC. T24-miR-NC or T24-miR-17-5p cells were pre- incubated with 1 mM NAC for 4 h and then treated with 24 nM or 50 nM paclitaxel for 24 h. Cell viability was measured by MTT assay. Data are represented as the mean ± S.E. (*p<0.05 vs. control, where n = 3).

The effect of miR-17-5p overexpression and subsequent paclitaxel treatment on the mitochondrial membrane potential of A549-T24 cells intrigued us to study the expression status of the major protein component of the mitochondrial apoptotic pathway. Paclitaxel treatment following miR-17-5p transfection for 24 h resulted in the increase in Bax (pro-apoptotic)/Bcl-2 (anti-apoptoic) ratio in A549-T24 cells accompanied by an increase in p53 level and increase in the amount of a 19 kDa caspase-3 cleavage intermediate as well as cleaved poly (ADP-ribose) polymerase (PARP) (Fig. 8A and Fig. S5A–C). We also assessed the relative caspase3 mRNA level by qRT- PCR and found overexpression of miR-17-5p and subsequent paclitaxel treatment resulted in increased caspase3 mRNA level compared to the negative control (Fig. 8B). Furthermore, to extend our finding we also measured mRNA expression levels of Bax, Bcl-2 and P53 proteins by qRT-PCR in H596-TxR cells following miR-17-5p overexpression. We found that overexpression of miR-17-5p and subsequent paclitaxel treatment resulted in significant upregulation of Bax and P53 mRNA expression (Fig. S5E and S5F) with a concomitant downregulation of Bcl-2 (Fig. S5D). Moreover, as shown in Fig. S6B, compared to the negative control (TxR-miR-NC) overexpression of miR-17-5p and subsequent treatment with paclitaxel resulted in upregulation of caspase3 mRNA level in TxR-miR-17-5p cells indicating increase in cellular apoptosis.

These results collectively suggested that autophagy inhibition by miR-17-5p overexpression and subsequent paclitaxel treatment resulted in induction of apoptotic cell death by caspase3 mediated pathway in paclitaxel resistant lung cancer cells.

### Overexpression of miR-17-5p Stimulates ROS Generation, Required for Paclitaxel Mediated Apoptosis

Recent studies have shown that involvement of reactive oxygen species (ROS) following paclitaxel treatment in the induction of autophagy and apoptosis and also demonstrated the importance of ROS in cytochrome-*c* release form the mitochondria to cytosol [14], [37]–[40]. So, we wanted to assess whether inhibition of autophagy by miR-17-5p overexpression and subsequent paclitaxel treatment could stimulate ROS generation in A549-T24 cells. T24-miR-NC and T24-miR-17-5p cells were treated with 24 nM and 50 nM paclitaxel for 24 h. The treated-cells were stained with cell-permeant fluorescent dye H2-DCFDA to detect the change in ROS generation. Fig. 8C and 8D shows that ROS levels were increased in T24-miR-17-5p cells compared to the respective negative control (T24-miR-NC) following paclitaxel treatment. We also noticed increased accumulation of ROS following miR-17-5p overexpression and paclitaxel treatment in H596-TxR cells (Fig. S6C). Furthermore, we were interested to examine whether ROS inhibition could influence paclitaxel mediated cell death. Therefore, we pre-treated both T24-miR-NC and T24-miR-17-5p cells with 1 mM N-acetyl-L- cysteine (NAC) for 4 h, followed by treatment with 24 nM or 50 nM paclitaxel for another 24 h after changing the NAC containing media and cell viability was measured by MTT assay. As shown in Fig. 8E, pre- treatment with NAC inhibited the cytotoxic effects of paclitaxel by scavenging ROS. Similar results were observed with TxR-miR-17-5p cells (Fig. S6D) where pre-treatment with 1 mM NAC and subsequent paclitaxel treatment resulted in increase in cell viability as measured by MTT assay. These results clearly indicated that inhibition of autophagy by miR-17-5p overexpression and subsequent paclitaxel treatment results in the generation of ROS which play a major role in regulating cell death response.

## Discussion

Autophagy, an evolutionarily conserved intra-cellular self defence mechanism, which not only prevent toxic accumulation of damaged cellular counterparts but is responsible for the degradation and recycling of cytoplasmic constituents including organelles, long-lived misfolded proteins to sustain metabolic homeostasis [13], [14], [31], [33]–[35], [41]. Previously many studies have reported stimulation of autophagy in response to treatment with chemotherapeutic agents including paclitaxel and internal cellular needs to maintain metabolism, which in turn contributed to development of chemo-resistance in many cancer types including NSCLCs [14], [33] and blocking cancer cell autophagy emerged as a novel approach to enhance the efficiency of chemotherapy in lung and other cancers [14], [33], [42], [43]. Moreover, in recent years our most current knowledge of molecular mechanism of cancer drug resistance began to include not only dysregulation of expression of protein coding genes but also that of non- coding regulatory RNA, especially miRNA [3], [23]–[25]. Previously, many groups have reported miRNA dysregulation following paclitaxel resistance in lung and other cancer types and tried to correlate particular miRNAs with the resistant phenotype. Here in this report, we were interested in investigating the role of miRNAs in regulating autophagy leading to paclitaxel resistance in lung non-small cancer cells. We performed miRNA arrays to compare the differential miRNA profiles of paclitaxel resistant and sensitive A549 cells (Fig. 1). We also demonstrated that *in vitro* generated paclitaxel resistant lung cancer cells (A549-T24 and H596-TxR) exhibited heightened level of autophagy compared to their paclitaxel sensitive counterparts (Fig. 2A–C and Fig. S2A–C). Our results clearly indicated that both beclin 1 and LC3-II which are considered as the most important autophagic markers were significantly upregulated in both A549-T24 and H596-TxR cells accompanied by the decrease in p62 expression in A549-T24 cells than parental cells (Fig. 2A–C and Fig. S2A–C).

From the array results we found that miR-17-5p was highly downregulated in A549-T24 cells (Fig. 1 and Fig. 2D). We also found significant reduction in expression of 13 other miRNAs. However, bioinformatics analysis using different target prediction tools and literature survey showed no direct involvement of these miRNAs in autophagic activity in lung cancer cells except for miR-106a and miR-101. But forced expression of these miRNAs into A549-T24 cells and subsequent paclitaxel treatment showed no significant change in paclitaxel sensitivity (Fig. S7). Elucidation of role of other miRNAs in paclitaxel resistance requires further study. Moreover, here we showed that miR-17-5p directly targeted beclin 1 in lung cancer cells and its overexpression resulting in downregulation of beclin 1 (Fig. 4 and Fig. 5 and Fig. S3B–D). Our results clearly indicated that inhibition of beclin-1 mediated autophagy by miR-17-5p overexpression significantly sensitized the paclitaxel resistant lung cancer cells to paclitaxel (Fig. 3 and Fig. S3A). Therefore, downregulation of BECN1 mediated cellular autophagy by miR-17-5p overexpression and subsequent paclitaxel treatment significantly increased the percentage of apoptotic cells in both A549-T24 and H596-TxR cells (Fig. 7A–B and Fig. S4) which was further confirmed by the change in expression of several pro- apoptotic and anti- apoptotic proteins (Fig. 8A–B and Fig. S5).

Paclitaxel was reported to affect mitochondrial apoptotic mechanism by altering mitochondrial permeability transition and changing the expression of several mitochondrial membrane proteins [2], [14], [33], [44], [45]. In this report, we also demonstrated that inhibition of cyto-protective autophagy by miR-17-5p overexpression and subsequent paclitaxel treatment caused change in mitochondrial membrane potential (Fig. 7C–D) and expression of mitochondrial apoptotic marker proteins (Fig. 8A–B and Fig. S5). Furthermore, recently many reports have shown that suppression of autophagy by silencing ATG7 and beclin 1 results in increase in ROS generation leading to sensitization of cancer cells to drug induced apoptosis [13], [14], [41]. So, we checked the role of ROS in paclitaxel induced cellular apoptosis in both A549-T24 and H596-TxR cells. We observed that inhibition of autophagy by miR-17-5p overexpression and subsequent paclitaxel treatment resulted in stimulated ROS formation and apoptotic cell death in A549-T24 cells (Fig. 8C–D and Fig. S6C). Moreover, inhibition of ROS accumulation by pre-treating cells with NAC following miR-17-5p overexpression inhibited paclitaxel induced cytotoxicity (Fig. 8E and Fig. S6D).

In summary, our results indicated that paclitaxel mediated cyto-protective autophagy was an important mechanism against apoptotic cell death in lung cancer cells and that gradually drove the cells to drug resistance. Here in this report, we demonstrated that miR-17-5p mediated regulation of autophagy by targeting BECN1 contributed to paclitaxel resistance in two lung cancer cell lines, A549 and H596 cells. Inhibition of autophagy by overexpression of miR-17-5p could be a novel strategy for combination chemotherapy of lung cancer. Moreover, we showed that increase in apoptosis, induced by autophagy inhibition was regulated by ROS generation suggesting that regulation of ROS generation and autophagy could provide a powerful strategy to overcome paclitaxel resistance in lung cancer and further study involving lung tumour samples is required.

## Supporting Information

Figure S1 **A549-T24 and H596-TxR cells show significantly lower sensitivity to paclitaxel.** (A) A549 and A549-T24 cells were seeded into 96 well plates at a density of 1×10^4^ cells per well. Cells were treated with 0–200 nM Paclitaxel for another 24 h. The cell viability was assessed by MTT assay. Data are presented as % of cell viability measured in cell treated with paclitaxel. *Columns*, mean of three independent experiments; *bars*, ± S.E. (B) H596 and H596-TxR cells were seeded into 96 well plates at a density of 1×10^4^ cells per well. Cells were treated with 0–200 nM Paclitaxel for another 24 h. The cell viability was assessed by MTT assay. Data are presented as % of cell viability measured in cell treated with paclitaxel. *Columns*, mean of three independent experiments; *bars*, ± S.E.
(TIF)

Figure S2 **H596-TxR cells exhibites heightened level of autophagy with downregulated miR-17-5p expression compared to paclitaxel sensitive H596 cells.** (A) Expression status of certain autophagic marker proteins BECN1, MAP-LC3 and GAPDH (loading control) were measured by Western blotting. (B–C) Relative BECN1 and LC3-II mRNA expression levels were quantified by qRT-PCR analysis in H596 and H596-TxR cells, *bars* represent mean ± S.E. (*p<0.001 and **p<0.03 vs control, where n = 4). (D) Downregulation of miR-17-5p expression in paclitaxel resistant H596-TxR compared to H596 cells. Taqman qRT-PCR was performed to detect the relative expression levels of miR-17-5p in H596 and H596-TxR cells. Results were normalized to snU6 expression level and represented as mean ± S.E. from three independent replicates. (*p<0.03 vs control, n = 3).
(TIF)

Figure S3 **miR-17-5p overexpression modulates paclitaxel response and inhibits BECN1 expression in H596-TxR cells.** (A) H596-TxR cells were either transfected with 100nM pre-miR-negative control (TxR-miR-NC) or pre-miR-17-5p (TxR-miR-17-5p) precursor RNA and were seeded into 96 well plates at a density of 1×10^4^ cells per well. After 24 h, cells were treated with 0–200 nM Paclitaxel for another 24 h. The cell viability was assessed by MTT assay. Data are presented as % of cell viability measured in cells treated with Paclitaxel. *Columns*, mean of three independent experiments; *bars*, mean ±S.E. (*p<0.03 vs control (H596 control and negative control), where n = 3). (B–D) miR-17-5p overexpression modulated BECN1 expression in H596-TxR cells. (B) H596-TxR cells were transfected either with 100 nM pre-miR-negative control (TxR-miR-NC) or pre-miR-17-5p (TxR-miR-17-5p) precursor RNA. After 24 h, cell lysates were prepared for Western blotting with antibody against BECN1, MAP-LC3 and GAPDH (loading control). (C–D) Relative BECN1 and LC3-II mRNA expression levels were quantified by qRT-PCR analysis in TxR-miR-NC and TxR-miR-17-5p cells, *bars* represent mean ± S.E. from three independent experiments (*p<0.03, **p<0.01 vs control, where n = 3).
(TIF)

Figure S4 **miR-17-5p overexpression and subsequent paclitaxel treatment induced apoptosis in H596-TxR cells.** (A) TxR-miR-NC or TxR-miR-17-5p cells were treated either with 24 nM or 50 nM paclitaxel for another 24 h. Cells were then harvested for apoptosis analysis by annexin V- FITC/PI staining and flowcytometry. The % of early apoptotic cells (annexin V-FITC positive/PI negative cells) and late apoptotic cells (annexin V-FITC positive/PI positive cells) were determined. The results represented are the best of data collected from three independent experiments with similar results. (B) Representation of % of apoptotic cells following pre-miRNA transfection and paclitaxel treatment.
(TIF)

Figure S5 **Measurement of relative mRNA levels of apoptotic marker proteins in A549-T24 and H596-TxR cells following miR-17-5p overexpression and subsequent paclitaxel treatment.** Relative expression levels of Bcl-2 (A), Bax (B) and P53 (C) mRNAs were quantified by qRT-PCR analysis in T24-miR-NC and T24-miR-17 cells after being treated with 24 nM and 50 nM paclitaxel for 24 h, *bars* represent mean ± S.E. from three independent experiments (*p<0.03, **p<0.01 vs control, n = 3). NC1, T1 represent T24-miR-NC and T24-miR-17-5p cells respectively. Similarly relative expression levels of Bcl-2 (D), Bax (E) and P53 (F) mRNAs were determined by qRT-PCR in TxR-miR-NC and TxR-miR-17 cells following treatment with 24 nM and 50 nM paclitaxel for 24 h, *bars* represent mean ± S.E. from three independent experiments (*p<0.03, **p<0.01 ***p<0.05 vs control, n = 3). NC2, T2 represent TxR-miR-NC and TxR-miR-17-5p cells respectively.
(TIF)

Figure S6 **Overexpression of miR-17 and subsequent paclitaxel treatment induced release of cytochrome-** ***c*** **from mitochondria to the cytosol and resulted in ROS generation in H596-TxR cells.** (A) Western blot analysis to detect the release of the cytochrome C in the cytosol from mitochondria in H596-TxR cells following miR-17-5p overexpression and subsequent paclitaxel treatment. (B) Relative caspase3 mRNA level was estimated by qRT-PCR in TxR-miR-17-5p cells compared to TxR-miR-NC cells following 24 nM or 50 nM paclitaxel treatment for 24 h, *bars* represent mean ± S.E. from three independent experiments (*p<0.03 vs control, n = 3). (C) miR-17-5p overexpression and subsequent paclitaxel treatment stimulated ROS generation in H596-TxR cells. TxR-miR-NC or TxR-miR-17-5p cells were treated with 50 nM paclitaxel for 24 h. ROS generation were estimated by staining the H2-DCFDA staining and flowcytometry. NC and T1 represent TxR-miR-NC cells treated with 50 nM paclitaxel and TxR-miR-17-5p cells treated with 50 nM paclitaxel respectively (D) Amelioration of paclitaxel induced cytotoxicity following miR-17-5p overexpression in H596-TxR cells by NAC. TxR-miR-NC or TxR-miR-17-5p cells were pre- incubated with 1 mM NAC for 4h and then treated with 50 nM paclitaxel for 24 h. Cell viability was measured by MTT assay. Data are represented as the mean ± S.E. (*p<0.03 vs. control, where n = 3).
(TIF)

Figure S7 **MTT assay.** A549-T24 cells were transfected either with 100 nM pre-miR-negative (T24-miR-NC) or pre-miR-101 (T24-miR-101) or pre-miR-106a (T24-miR-106a) and were seeded into 96 well plates at a density of 1×10^4^ cells per well. Then cells were treated with 0, 12, 24, 50, 100, 200 nM paclitaxel for another 24 h. The cell viability was assessed by MTT assay. Data are presented as % of cell viability measured in cell treated with paclitaxel. *Columns*, mean of three independent experiments; *bars*, ± S.E.
(TIF)
