# Supplementary figures and images for: miR-17-5p Downregulation Contributes to Paclitaxel Resistance of Lung Cancer Cells through Altering Beclin1 Expression
Source: PLoS One. 2014 Apr 22;9(4):e95716. doi: 10.1371/journal.pone.0095716 (PMC3995800; doi:10.1371/journal.pone.0095716)

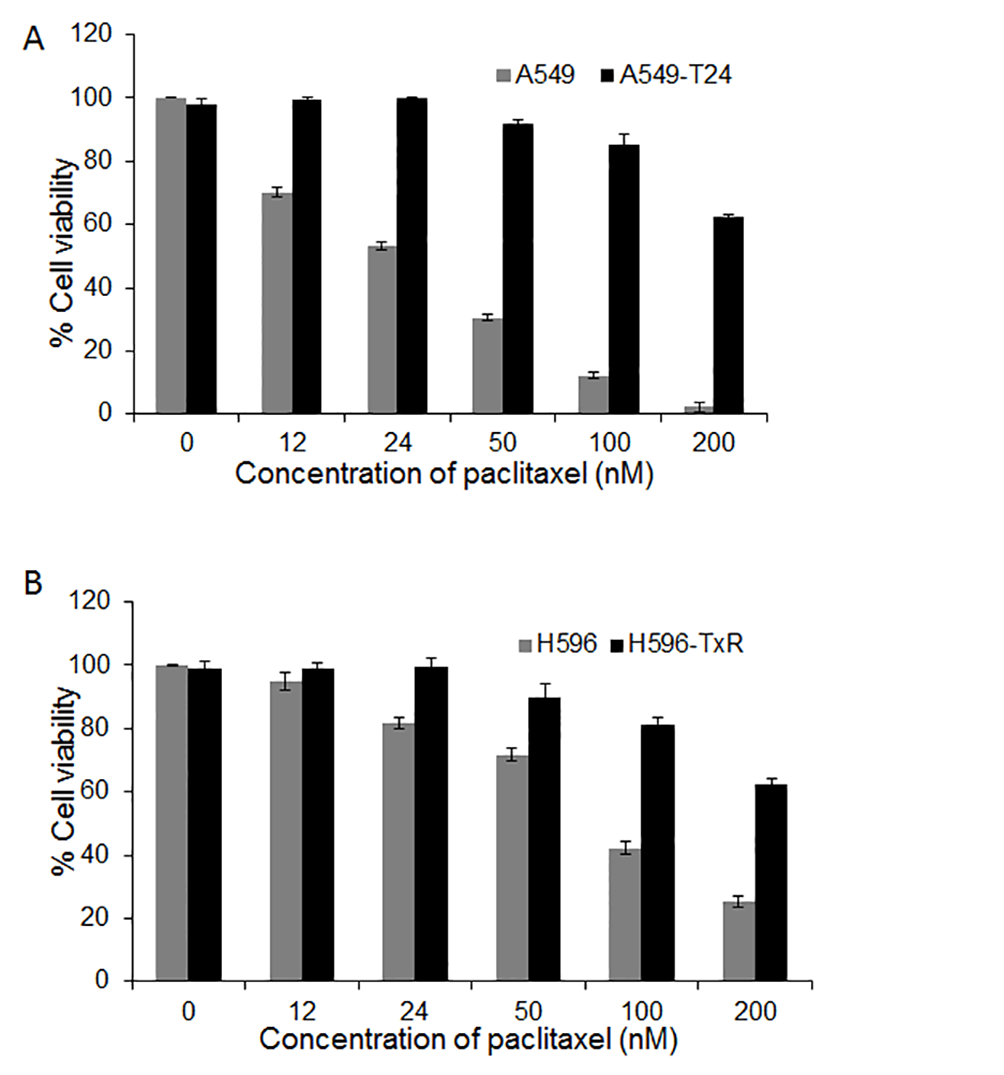

Supplement: Figure S1 — A549-T24 and H596-TxR cells show significantly lower sensitivity to paclitaxel. (A) A549 and A549-T24 cells were seeded into 96 well plates at a density of 1×10^4^ cells per well. Cells were treated with 0–200 nM Paclitaxel for another 24 h. The cell viability was assessed by MTT assay. Data are presented as % of cell viability measured in cell treated with paclitaxel. Columns, mean of three independent experiments; bars, ± S.E. (B) H596 and H596-TxR cells were seeded into 96 well plates at a density of 1×10^4^ cells per well. Cells were treated with 0–200 nM Paclitaxel for another 24 h. The cell viability was assessed by MTT assay. Data are presented as % of cell viability measured in cell treated with paclitaxel. Columns, mean of three independent experiments; bars, ± S.E. (TIF) [file pone.0095716.s001.tif]

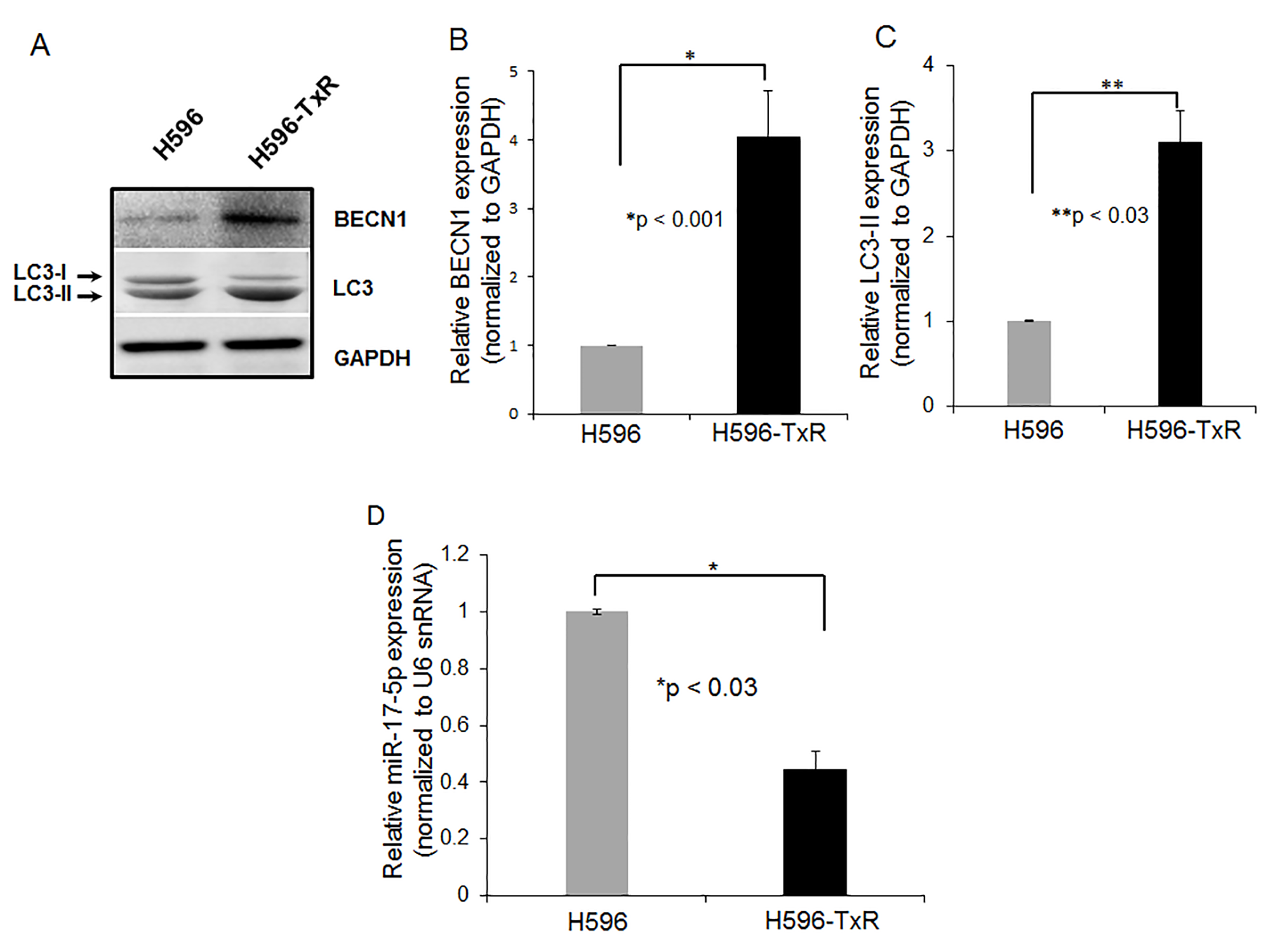

Supplement: Figure S2 — H596-TxR cells exhibites heightened level of autophagy with downregulated miR-17-5p expression compared to paclitaxel sensitive H596 cells. (A) Expression status of certain autophagic marker proteins BECN1, MAP-LC3 and GAPDH (loading control) were measured by Western blotting. (B–C) Relative BECN1 and LC3-II mRNA expression levels were quantified by qRT-PCR analysis in H596 and H596-TxR cells, bars represent mean ± S.E. (*p<0.001 and **p<0.03 vs control, where n = 4). (D) Downregulation of miR-17-5p expression in paclitaxel resistant H596-TxR compared to H596 cells. Taqman qRT-PCR was performed to detect the relative expression levels of miR-17-5p in H596 and H596-TxR cells. Results were normalized to snU6 expression level and represented as mean ± S.E. from three independent replicates. (*p<0.03 vs control, n = 3). (TIF) [file pone.0095716.s002.tif]

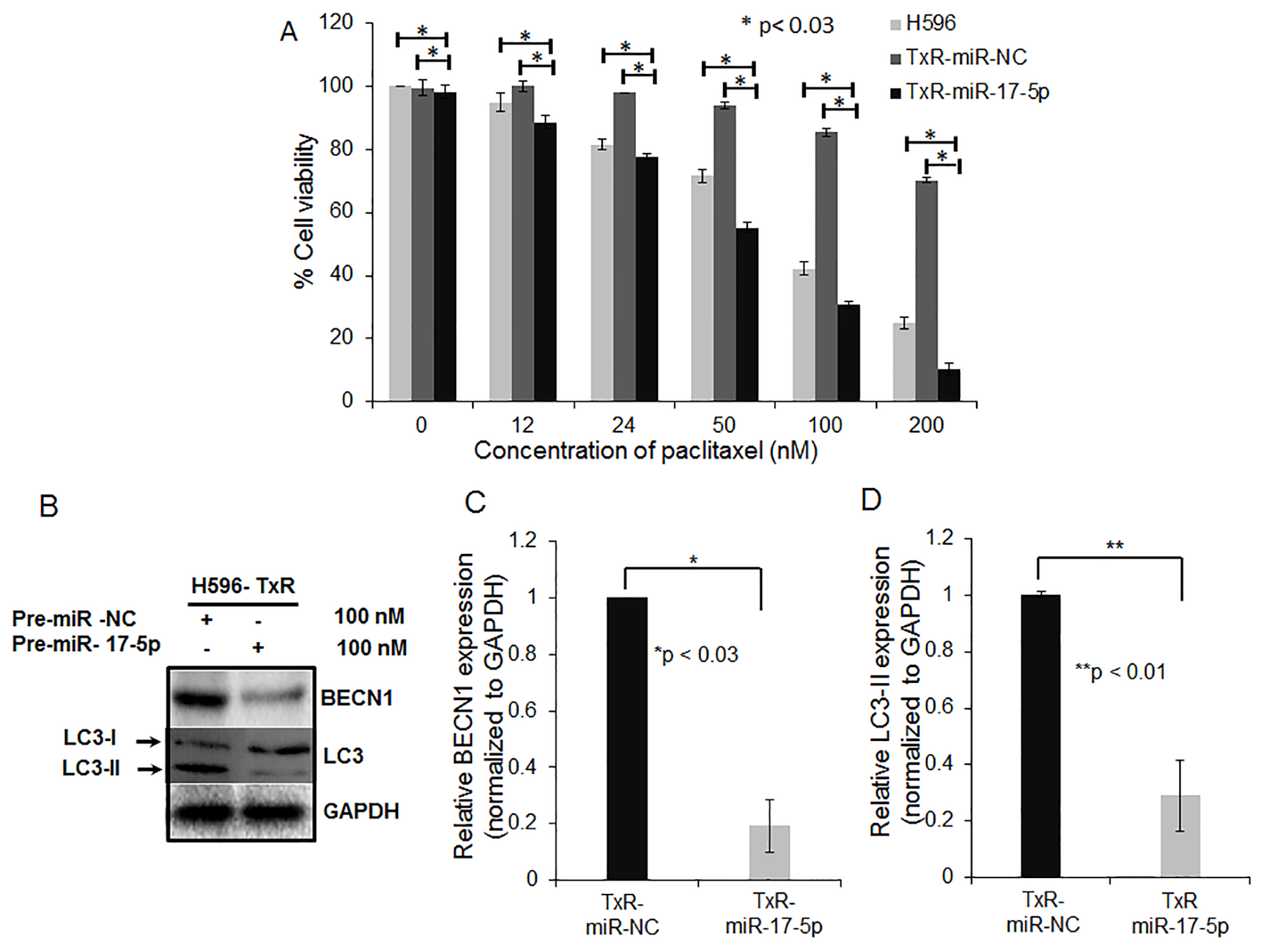

Supplement: Figure S3 — miR-17-5p overexpression modulates paclitaxel response and inhibits BECN1 expression in H596-TxR cells. (A) H596-TxR cells were either transfected with 100nM pre-miR-negative control (TxR-miR-NC) or pre-miR-17-5p (TxR-miR-17-5p) precursor RNA and were seeded into 96 well plates at a density of 1×10^4^ cells per well. After 24 h, cells were treated with 0–200 nM Paclitaxel for another 24 h. The cell viability was assessed by MTT assay. Data are presented as % of cell viability measured in cells treated with Paclitaxel. Columns, mean of three independent experiments; bars, mean ±S.E. (*p<0.03 vs control (H596 control and negative control), where n = 3). (B–D) miR-17-5p overexpression modulated BECN1 expression in H596-TxR cells. (B) H596-TxR cells were transfected either with 100 nM pre-miR-negative control (TxR-miR-NC) or pre-miR-17-5p (TxR-miR-17-5p) precursor RNA. After 24 h, cell lysates were prepared for Western blotting with antibody against BECN1, MAP-LC3 and GAPDH (loading control). (C–D) Relative BECN1 and LC3-II mRNA expression levels were quantified by qRT-PCR analysis in TxR-miR-NC and TxR-miR-17-5p cells, bars represent mean ± S.E. from three independent experiments (*p<0.03, **p<0.01 vs control, where n = 3). (TIF) [file pone.0095716.s003.tif]

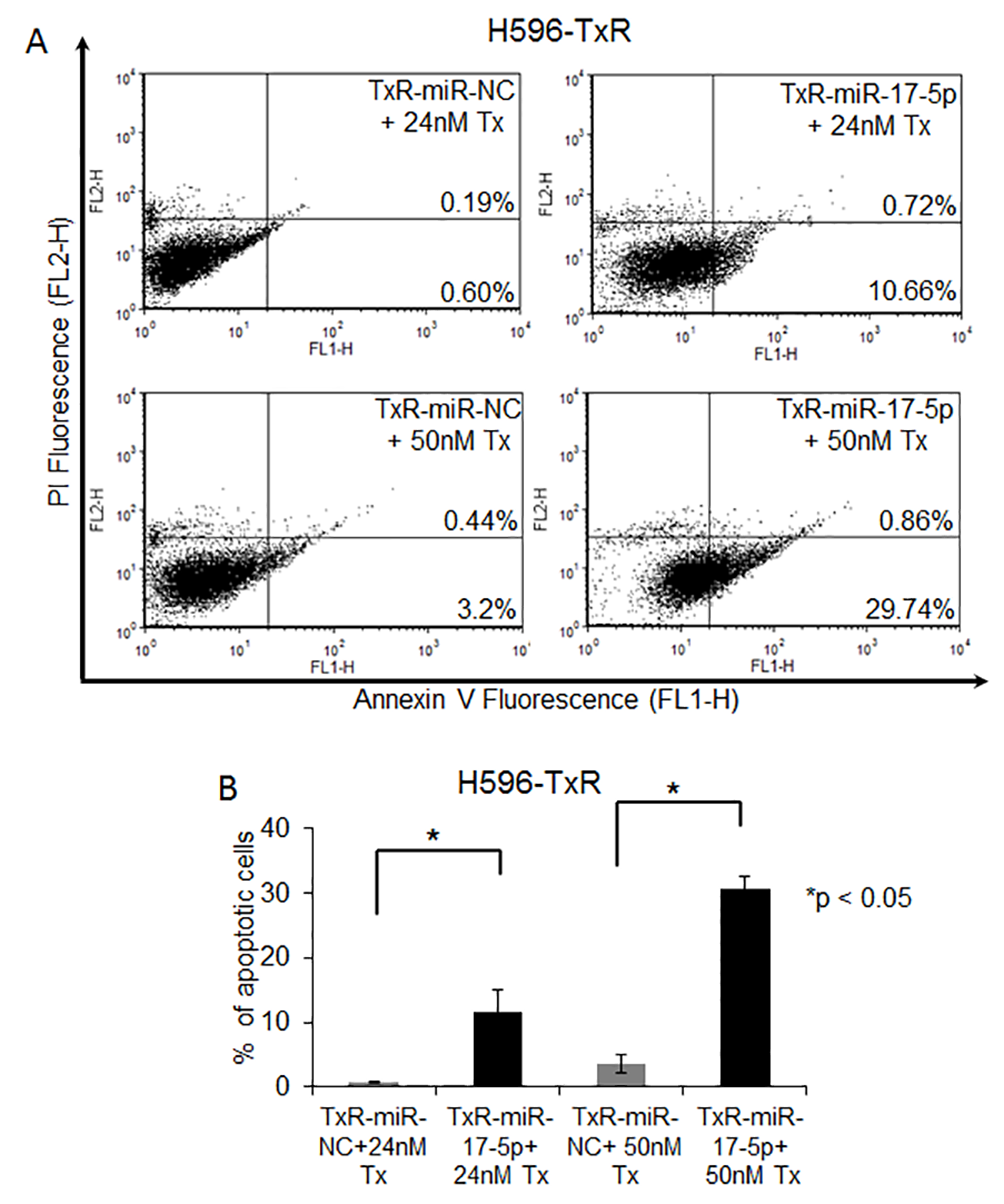

Supplement: Figure S4 — miR-17-5p overexpression and subsequent paclitaxel treatment induced apoptosis in H596-TxR cells. (A) TxR-miR-NC or TxR-miR-17-5p cells were treated either with 24 nM or 50 nM paclitaxel for another 24 h. Cells were then harvested for apoptosis analysis by annexin V- FITC/PI staining and flowcytometry. The % of early apoptotic cells (annexin V-FITC positive/PI negative cells) and late apoptotic cells (annexin V-FITC positive/PI positive cells) were determined. The results represented are the best of data collected from three independent experiments with similar results. (B) Representation of % of apoptotic cells following pre-miRNA transfection and paclitaxel treatment. (TIF) [file pone.0095716.s004.tif]

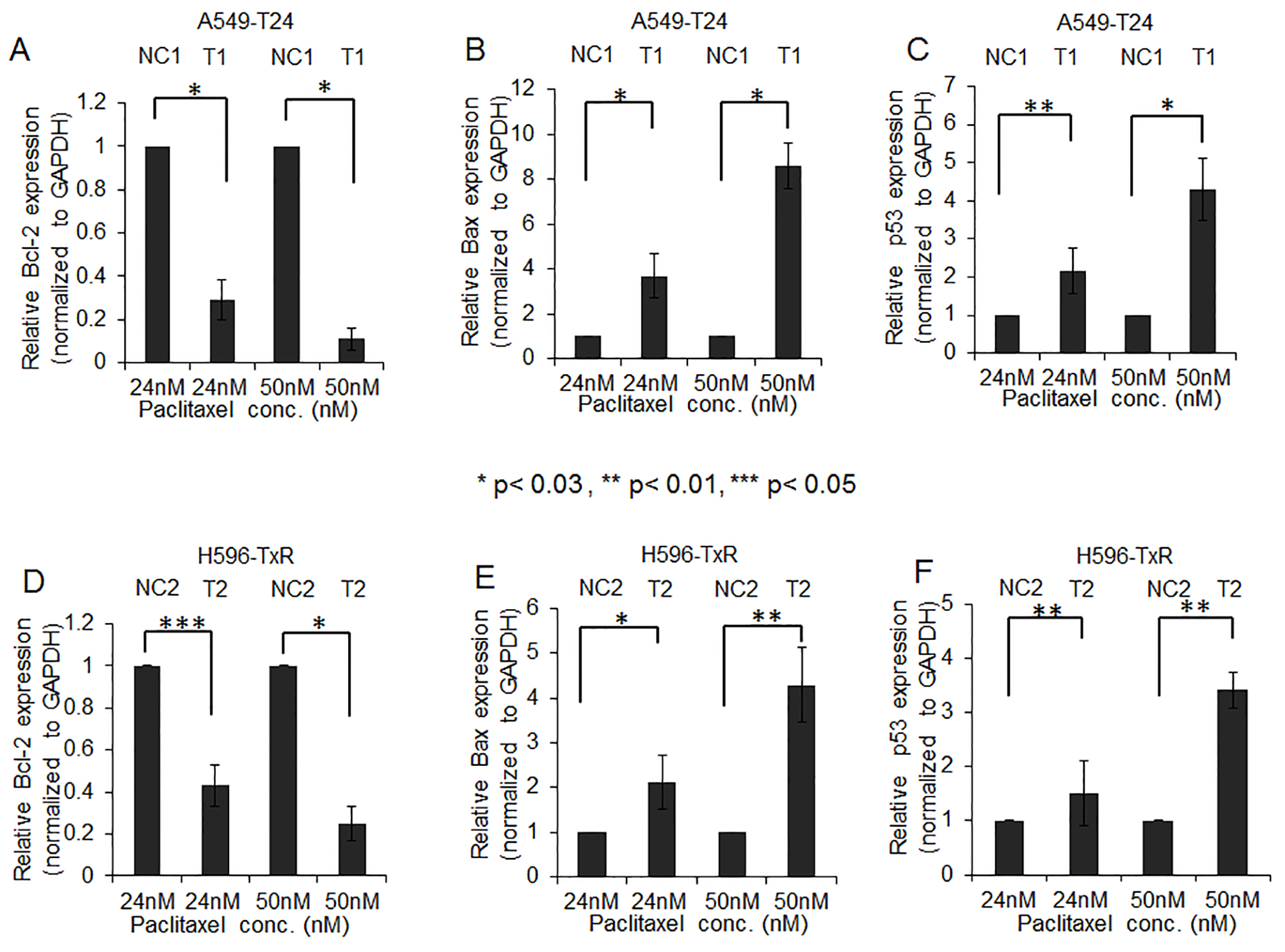

Supplement: Figure S5 — Measurement of relative mRNA levels of apoptotic marker proteins in A549-T24 and H596-TxR cells following miR-17-5p overexpression and subsequent paclitaxel treatment. Relative expression levels of Bcl-2 (A), Bax (B) and P53 (C) mRNAs were quantified by qRT-PCR analysis in T24-miR-NC and T24-miR-17 cells after being treated with 24 nM and 50 nM paclitaxel for 24 h, bars represent mean ± S.E. from three independent experiments (*p<0.03, **p<0.01 vs control, n = 3). NC1, T1 represent T24-miR-NC and T24-miR-17-5p cells respectively. Similarly relative expression levels of Bcl-2 (D), Bax (E) and P53 (F) mRNAs were determined by qRT-PCR in TxR-miR-NC and TxR-miR-17 cells following treatment with 24 nM and 50 nM paclitaxel for 24 h, bars represent mean ± S.E. from three independent experiments (*p<0.03, **p<0.01 ***p<0.05 vs control, n = 3). NC2, T2 represent TxR-miR-NC and TxR-miR-17-5p cells respectively. (TIF) [file pone.0095716.s005.tif]

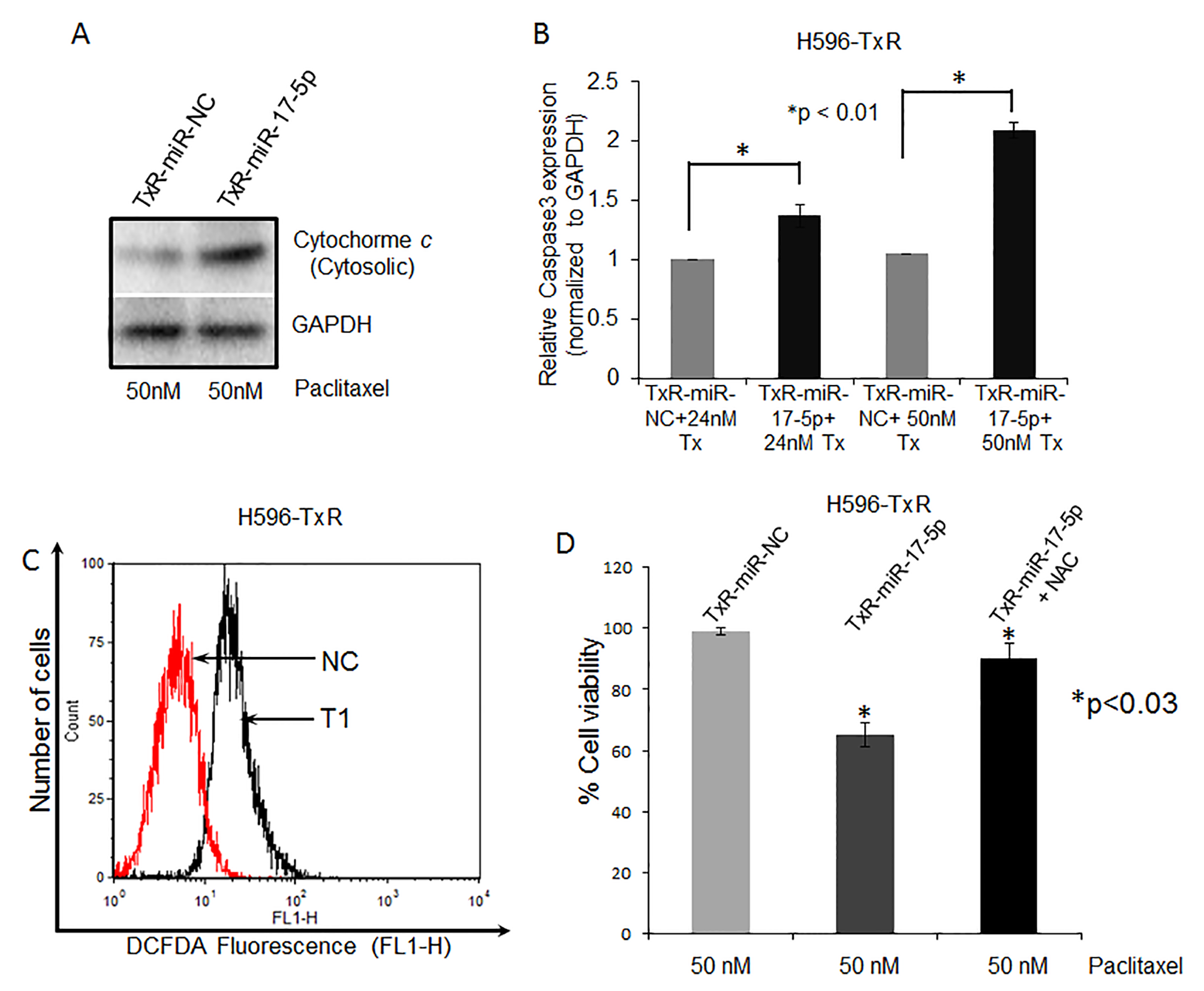

Supplement: Figure S6 — Overexpression of miR-17 and subsequent paclitaxel treatment induced release of cytochrome- c from mitochondria to the cytosol and resulted in ROS generation in H596-TxR cells. (A) Western blot analysis to detect the release of the cytochrome C in the cytosol from mitochondria in H596-TxR cells following miR-17-5p overexpression and subsequent paclitaxel treatment. (B) Relative caspase3 mRNA level was estimated by qRT-PCR in TxR-miR-17-5p cells compared to TxR-miR-NC cells following 24 nM or 50 nM paclitaxel treatment for 24 h, bars represent mean ± S.E. from three independent experiments (*p<0.03 vs control, n = 3). (C) miR-17-5p overexpression and subsequent paclitaxel treatment stimulated ROS generation in H596-TxR cells. TxR-miR-NC or TxR-miR-17-5p cells were treated with 50 nM paclitaxel for 24 h. ROS generation were estimated by staining the H2-DCFDA staining and flowcytometry. NC and T1 represent TxR-miR-NC cells treated with 50 nM paclitaxel and TxR-miR-17-5p cells treated with 50 nM paclitaxel respectively (D) Amelioration of paclitaxel induced cytotoxicity following miR-17-5p overexpression in H596-TxR cells by NAC. TxR-miR-NC or TxR-miR-17-5p cells were pre- incubated with 1 mM NAC for 4h and then treated with 50 nM paclitaxel for 24 h. Cell viability was measured by MTT assay. Data are represented as the mean ± S.E. (*p<0.03 vs. control, where n = 3). (TIF) [file pone.0095716.s006.tif]

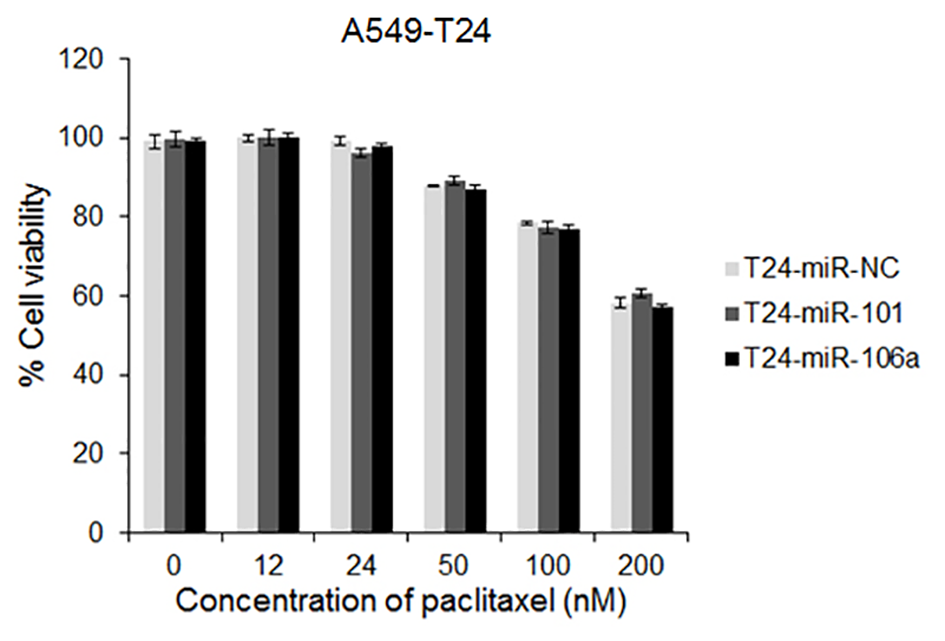

Supplement: Figure S7 — MTT assay. A549-T24 cells were transfected either with 100 nM pre-miR-negative (T24-miR-NC) or pre-miR-101 (T24-miR-101) or pre-miR-106a (T24-miR-106a) and were seeded into 96 well plates at a density of 1×10^4^ cells per well. Then cells were treated with 0, 12, 24, 50, 100, 200 nM paclitaxel for another 24 h. The cell viability was assessed by MTT assay. Data are presented as % of cell viability measured in cell treated with paclitaxel. Columns, mean of three independent experiments; bars, ± S.E. (TIF) [file pone.0095716.s007.tif]
